# Spatial early warning signals to assess economic resilience

**DOI:** 10.1016/j.isci.2025.114097

**Published:** 2025-11-17

**Authors:** Sol Maria Halleck Vega, Roberto Patuelli, George van Voorn, Els Weinans

**Affiliations:** 1 Urban Economics, Wageningen University & Research, Wageningen, the Netherlands; 2 Department of Economics, University of Bologna, Bologna, Italy; 3 The Rimini Centre for Economic Analysis, Italy; 4 Biometris, Wageningen University & Research, Wageningen, the Netherlands; 5 Copernicus Institute of Sustainable Development, Utrecht University, Utrecht, the Netherlands

**Keywords:** Geography, Economics, Human Geography

## Abstract

Improving the resilience of economies to crises is of societal and policy interest. In this article, we complement the regional economics literature on resistance and recovery facets of resilience by instead exploring signals for loss of resilience prior to crises. In particular, we adapt spatiotemporal indicators from the ecological literature to spatially disaggregated unemployment data to assess the resilience of the economy of France. Key questions are whether more information about the resilience of the system can be gained by considering the spatial dimension, and whether the indicators can be used as a detection method of impending economic crises. This approach reveals that a spatially disaggregated principal components analysis enables to capture of signals of critical slowing down and to assess which specific region or groups of regions dominate the unemployment dynamics, which represents critical information that is missed when using nonspatial early warning signals. We find that different regions dominate the signal before or after a crisis. This resembles response diversity as seen in ecosystems. The spatial early warning signal, Moran’s I, is found to increase prior to the moments of economic crises. These findings suggest that the spatial characteristics of a country’s unemployment are crucial to assess a country’s resilience.

## Highlights

• Resilience assessment for economic systems improves when considering spatial data
• We propose an approach that indicates loss of resilience prior to crises
• Spatial signal (Moran’s I) can provide early warnings before economic crises
• Principal component analysis helps to identify regions dominating the signal

## Graphical abstract

**Figure:**
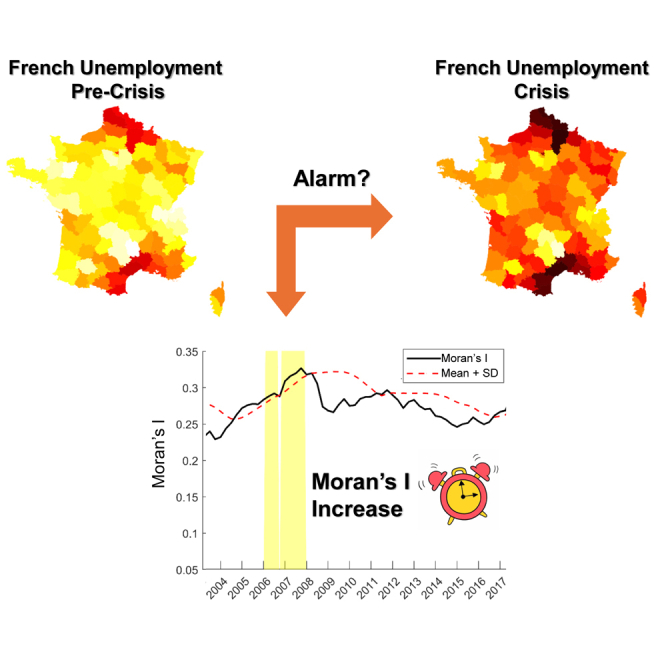

## Introduction

Economies are subject to crises that can have profound impacts on societies, such as on labor market performance. Resilience - the capacity of a system to maintain or restore its functioning after a shock^1^ - against such crises is of obvious interest. Resilience has increasingly gained attention in regional economics in the wake of the 2008 financial crisis, with seminal work on how regional economies respond to recessionary shocks.^2^^,^^3^ Studies in this literature strand usually focus on two facets of resilience: resistance and recovery – i.e., the ability of regions to resist the initial impact of a shock and recover after the crisis.^4^ The conceptualization of regional economic resilience thus shares some similarities to that of socio-ecological systems. Resilience is specific (“resilience of what, to what?”^5^; “five W’s”^6^): some crises have been caused by shocks that are exogenous from the viewpoint of financial systems such as the Covid-19 pandemic, while others have been caused by endogenous processes, such as the 2008 financial crisis.^7^ Understanding which mechanisms are at work is a necessary first step in improving resilience.^8^^,^^9^ While aggregate measures are typically used in economic studies, such as national GDP and unemployment, which often seem to correlate well with economic crisis moments, such data are of limited help in ascertaining essential mechanisms behind resilience against these crises. Finer grained information may be required to quantify how different elements in the system may interact with each other, and how these interactions may give rise to stabilizing or destabilizing processes that can either prevent or cause the crashing of an economic system.^10^^,^^11^

To understand these (de)stabilizing mechanisms, economists have recently started to look to complexity science for answers,^11^ and in particular to theory on resilience mechanisms.^12^ Economic systems can be viewed as complex systems^13^^,^^14^^,^^15^ that include endogenous processes involving interactions between actors at different (spatial) scales, such as individuals, firms, banks, and governments.^7^^,^^10^^,^^12^ A fundamental property of such “complex systems” is that their macroscopic behavior arises from the interactions of the actors at smaller scales. For this reason, the proposed premise here is that disaggregated data could aid the identification of resilience mechanisms.

**Figure 1:**
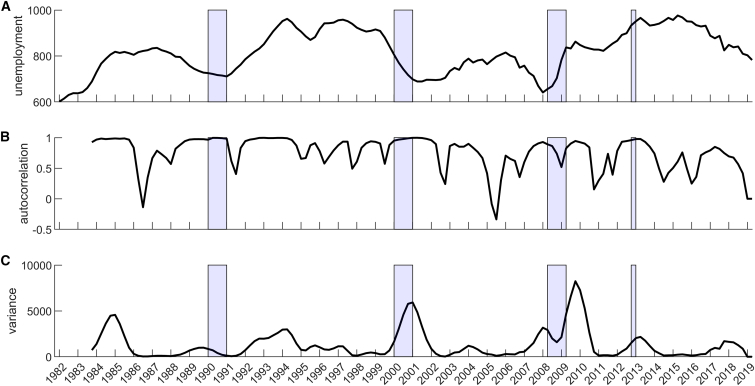
No concise signal is found to predict French recession periods (shaded areas) with a univariate analysis of the aggregated unemployment data (A) Aggregate unemployment data (unemployment rates summed over all departments). (B) Autocorrelation of the aggregated unemployment signal. C Variance of the aggregated unemployment signal.

In this article, we adapt methods from the ecological literature focusing on indicators for loss of resilience before a “tipping point.” Several recent studies provide evidence that economic crises may be manifestations of tipping points.^14^^,^^16^^,^^17^ Mathematically, a tipping point is associated with system conditions in which structural changes occur in the system that result from complex interactions, for example, a stable system state ceases to exist (i.e., can no longer be maintained). In other words, nearing the tipping point the resilience of the system has been eroded, which means that any perturbation will automatically “push” the system to an alternative state.^18^ Resilience indicators, also known as early warning signals - EWS for detecting tipping points, that have been developed mostly in the ecological literature have proved useful to explore financial crises in several studies.^17^^,^^19^^,^^20^^,^^21^^,^^22^ Two such EWS, autocorrelation and variance, capture how quickly a system recovers from a perturbation: nearing a tipping point, this recovery rate can decrease, a phenomenon also known as “critical slowing down,” and that, as such, represents the system’s resilience. A caveat is that financial systems differ from ecological systems, and signals using EWS may not show as clearly as for some ecological cases in controlled environments, where complexity is limited because many interactions are eliminated by the experimental set-up.^23^^,^^24^ This is also evident from the example of France, for which Figure 1 shows these typical EWS (autocorrelation and variance), but these indicators do not display a clear signal prior to the economic crises.

A promising line of work on ecological resilience indicators explicitly considers the spatial characteristics of a system.^25^^,^^26^ After the 2008 financial crisis, an acknowledgment developed that the observations at the macro level are a result of the interplay between several subsystems at a smaller level.^7^^,^^10^ A key question in this article is whether we gain more information on the resilience of an economic system by applying a spatial EWS (SEWS) approach, i.e., considering changes in spatial characteristics of a system that could provide early warnings of approaching transitions. SEWS relate to the literature on the relationship between networks, such as the density of connections and resilience to shocks.^12^^,^^27^^,^^28^^,^^29^ In Moro et al. (2015), an analogy is made to models of ecological mutualism, where labor markets in cities that employ workers in occupations with similar skill requirements (i.e., greater job connectivity) increases cities’ economic resilience.^12^ Besides occupational mobility acting as an adjustment mechanism, geographical labor (im)mobility can be a (lack of a) coping mechanism to a shock. People and processes between sectors are linked, and sectors may correlate to spatial orientation (e.g., tourism in the South and heavy industry in the North), while people working in those sectors will be limited in their operational range (traveling time, social networks, neighborhood affinity, and so forth). It seems reasonable to assume that such spatial aspects should be considered to obtain a clearer signal with an EWS indicator.

Here, we further explore whether changing spatial characteristics of labor markets can provide information on the economic resilience mechanisms within a country. The focus is on unemployment, a key indicator of the state of the economy, which often moves in tandem with crisis moments. The empirical application is for France. Importantly, this allows us to work with a high-quality dataset in terms of the numerosity of regions and time periods, which is ideal for analyzing time series of regional unemployment data. It also makes for an interesting case, as despite its social protection system, France has also gone through downturns, such as in the aftermath of the 2008 financial crisis. Regional unemployment rates tend to exhibit a strong correlation both over time and across space.^30^^,^^31^^,^^32^^,^^33^ While some spatial economic and econometric studies implement spatial autocorrelation as interaction terms in a model,^34^ other studies carried out initial exploratory analyses on spatial patterns in the economy,^35^^,^^36^ but the interpretation of those spatial patterns remains challenging.

We hypothesize that these spatial correlations provide relevant information on the resilience of a country’s economy, in line with ecological studies that indicate that spatial patterns provide information on the resilience of an ecosystem.^25^^,^^37^^,^^38^^,^^39^ Thus far, no link has been drawn between SEWS and the regional economics and spatial econometrics literature. In this way, we aim to complement more traditional economic-theoretical, economic geography, and regional economic literature on the topic, encompassing analyses of cyclical sensitivity, resistance, and recovery to the impacts of economic shocks.^40^^,^^41^^,^^42^^,^^43^^,^^44^ In particular, economic geographers found evidence of leads and lags among regional unemployment series over economic cycles (i.e., differences in sensitivity of regional unemployment to aggregate fluctuations) - this literature was most popular from the 1960s–80s, but has gained more recent attention considering the prevalence of recessionary shocks.^33^^,^^40^^,^^41^^,^^45^ In addition, as aforementioned, regional economists have also contributed to analyzing the resistance and recovery aspects of resilience via different measures, such as via a sensitivity index comparing the percentage change in employment in a given region with the change at the national level after a recessionary shock.^3^^,^^4^^,^^46^ By taking an alternative approach inspired by the early warning and SEWS work from ecology to improve our understanding of the resilience of national economies to crises, we thus complement the work of regional economists and economic geographers on this topic. This study also relates to so-called “intranational macroeconomics,” highlighting that information on the behavior of regions making up a macroeconomy can provide insights into the temporal dynamics of that economy and, accordingly, relevant macroeconomic policy insights.^47^^,^^48^ We perform a principal component analysis (PCA) inspired by Lever et al. (2020)^49^ and calculate the spatial autocorrelation as an SEWS inspired by Dakos et al. (2010)^50^ on our subnational unemployment data to uncover the resilience mechanisms that play a role in the economy. Furthermore, we assess the quality of Moran’s I à la Quax et al. (2013),^19^ as an indicator in signaling impending economic crises.

## Results

To highlight the utility of spatiotemporal ecological indicators for resilience research in economics, in our analyses, we use quarterly unemployment rates from Q1 1982 to Q2 2019 at the NUTS-3 level of geographical aggregation. In particular, the sample covers the 96 departments in metropolitan France.

**Figure 2:**
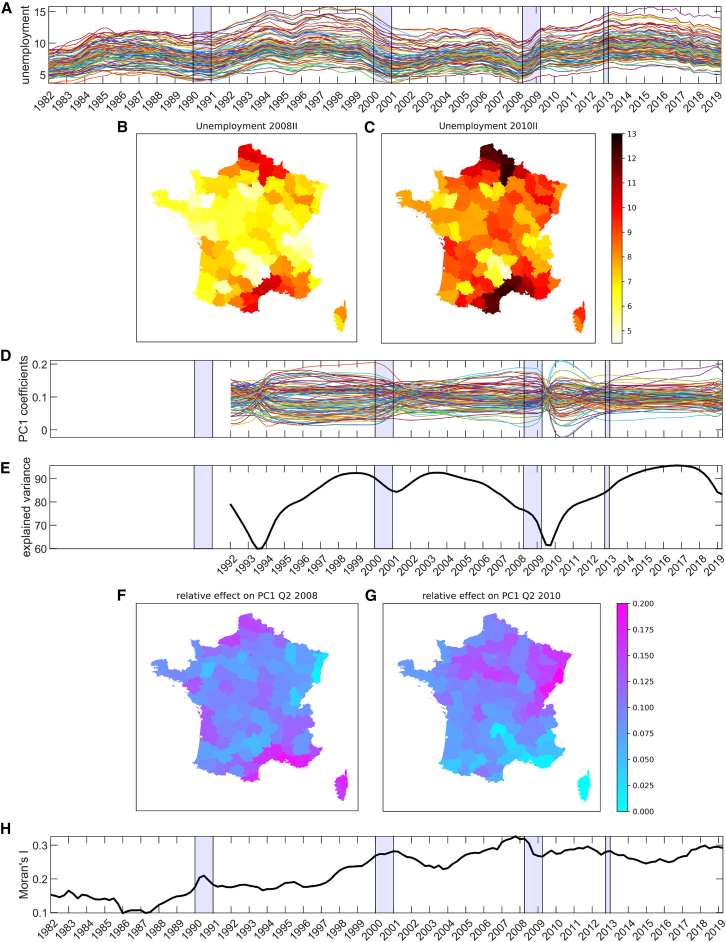
Ecological spatiotemporal resilience for the metropolitan departments of France (A) Unemployment rates (in percentage), where each colored line represents a French department. Shaded bars indicate crisis moments. (B and C) Maps of unemployment rates in Q2 2008 and Q2 2010. (D) First PC coefficients calculated as factor loadings multiplied by explained variance based on PCA, moving window = 10 years. (E) Plot of explained variance (in percentage) calculated on the moving window analysis. (F and G) Maps visualization of the first PC coefficients (same as in 2D), visualized spatially at the onset and after the 2008 crisis. (H) Moran’s I calculation as a spatial early warning signal based on critical slowing down.

First, it is apparent that the broad movement of the unemployment rates of all departments corresponds quite well to the major economic crisis years (Figure 2A). In particular, they mostly rose around the mid-1990s and then again following the 2008 global financial crisis (recession from Q2 2008 to Q2 2009). The economy again went into a downturn in 2012 (recession from Q4 2012 to Q1 2013), despite the French social protection system aimed at mitigating the impact of the crisis.^51^ Within the broad similarities in temporal patterns, it is also evident from Figure 2A that there are variations across departments. These spatial differences are striking; there are departments with higher unemployment in the north and the south. These departments coincide with regions that are less developed, and where the economy is more touristic/seasonal (see Figures 2B and 2C).

To unravel the dominant fluctuations within the unemployment data, we use PCA on the regional unemployment data. Lever et al. (2020) demonstrated that the large fluctuations that are picked up by PCA correspond to variables (in our case, regions) with low resilience (see the Methods section for details).^49^ PCA shows an inversion of departments in between the two crisis moments following 2008 (Figure 2D). This indicates a response to the crisis, where there is a shift of departments that determine the first principal component (PC1), i.e., the regions that dominate the dynamics (Figure 2D). During this shift, the explained variance drops as the PC1 is turning toward the new post-crisis situation (Figure 2E). This dip in explained variance is also evident from previous economic downturns, albeit the most prominent is observed in the vicinity of the 2008 crisis. By plotting the PC1 coefficients on the map, we can observe that there is a change in the ranking of the departments that dominate the unemployment signal before and after the crisis (Figures 2F and 2G). In particular, the unemployment in the mid-south and southeast regions played a leading role in the onset of the crisis, in contrast to that which built up after the crisis in the northeastern part of France. Interestingly, while most are border areas, Paris is an exception at the start of the crisis, as it also shows a high score on the first PC, but then does not appear to be as relevant in explaining changes in employment (see Figure 2G). Also, more inland areas in the center-north, and especially departments bordering Germany and Switzerland, dominate the post-crisis unemployment dynamics, indicated by their high score on the first PC (Figure 2G).

These results highlight that more information is gained on the economic system and its resilience via the spatial disaggregation of the PCA to capture signals of critical slowing down, i.e., whether a system is losing resilience (see the Methods section), compared to univariate methods. In particular, we can observe which specific region or groups of regions guide temporal patterns of unemployment. In this case, we find substantial changes in the relevance of different French departments in guiding these unemployment dynamics. Structural differences between areas, such as concentrations of certain types of industries in regions, are a main reason for spatial imbalances in labor demand.^4^^,^^52^ Economic sectors and how they are divided across a country could thus be an important resilience mechanism. The observed phenomenon that the regions that drive the unemployment dynamics after a crisis are different from the regions that drive the unemployment dynamics before the crisis may be comparable to response diversity observed in ecosystems; for example, species diversity enables resilience, because if one species disappears, another species can take over the ecological niche of the extinct species.^53^^,^^54^

Besides the information gained via the spatial disaggregated PCA, it is germane to explore whether further insights on the resilience of economic systems can be obtained by applying SEWS. To evaluate this, we turn to the results à la Kéfi et al. (2014) (see the Methods section).^25^ Moran’s I shows an overall upward trend, indicating that unemployment becomes more spatially clustered over time (see Figure 2H). This makes it hard to assess whether an increasing Moran’s I is indicative of an approaching crisis. However, the strength of the analysis is that it demonstrates that spatial structures are present and may change over time, and thus are a potential source of information about the economic functioning of a country. Furthermore, it could be the case that spatial autocorrelation represents resilience not because of a critical slowing down, but simply because if region A has high unemployment, but neighboring region B has a low unemployment, citizens of region A will find work in region B, and in that sense quickly recover from the high unemployment situation, whereas when region A and B both have a high unemployment, larger (systemic) transformation is required (e.g., either new industries in the regions, or migration).

If there is a lack of coping mechanisms, such as in Moro et al. (2021) via skill similarity, migration, or labor force participation adjustment, there can indeed be more regional unemployment polarization.^12^^,^^55^ Thus, if such mechanisms are absent, this can result in an amplification of spatial clustering. Furthermore, if an area contains high unemployment among workers with certain skills and badly performing industries, this can have negative spillover effects on other industries in the area, such as services and intermediate goods industries, via inter-industry linkages.^4^ The spatial imbalances in demand resulting from structural differences between areas can thus result in more pronounced unemployment clusters in the vicinity of a transition such as the 2008 crisis.

**Figure 3:**
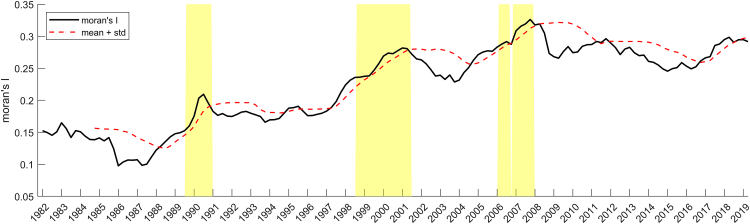
Moran’s I behavior and crisis moments for France The “alarm” shown in yellow is when: (A) Moran’s I is higher than its mean over the past 3 years + its standard deviation (i.e., the black line in the figure is above the red dotted line), and (B) the Kendall tau correlation of the past 3 years’ Moran’s I correlated to [1,2,3, …,12] is higher than 0.9, and (C) the *p*-value for the Kendall tau correlation above is lower than 0.05.

To further check if the Moran’s I dynamics are related to the crisis moments, we quantitatively assess the quality of this SEWS indicator using three criteria: there is a warning when (a) Moran’s I is larger than its mean +standard deviation over the past three years, and (b) the Kendall tau correlation is higher than 0.9, and (c) the *p*-value for the Kendall tau correlation is lower than 0.05 in line with Quax et al. (2013) and Dakos et al. (2010) (see the Methods section for details).^19^^,^^50^ From Figure 3 it emerges that Moran’s I indicator indeed precedes a crisis, i.e., it increases before a transition (high true positive rate) and does not significantly increase when there is no transition (low false positive rate). This is especially the case for the 2008 crisis; for the first two crises, the yellow shaded areas are also clearly linked and overlap the crisis moments.

We also test the sensitivity of this outcome for a range of parameters, namely varying window sizes, values multiplied by the standard deviation, and Kendall tau correlation threshold (see Supplementary Note 1). The sensitivity analysis reveals that a small window size leads to an increase in false positives (Figure S1). If we adapt criterion (a), and only warn when the signal is higher than the mean +1.5 times the standard deviation, all warnings emerge later and have a shorter duration. With 2 times the standard deviation, the analysis misses the 2000 and 2008 crises (increased false negative rate) (Figure S2). Lastly, when we change criterion (b), and only warn when the Kendall tau correlation is higher than 0.8, we see some false positives emerge. For increasing the Kendall tau correlation, our findings do not deviate from the default values (Figure S3). Thus, despite some differences, most parameter values corroborate the utility of Moran’s I as a tool to help better understand signals of impending economic crises.

We find that, like in ecological systems, response diversity seems to be relevant to explain economic resilience. In social systems, trade from multiple sources can boost resilience, as well as having a high-diversity system such as an agroecosystem compared to a monoculture.^54^ Economies with a more diversified sector composition tend to have a higher ability to adapt to changing circumstances.^42^^,^^43^^,^^52^ In particular, when there is a higher dependence on manufacturing and construction, economies tend to be less resilient due to higher elasticity of demand.^4^^,^^44^^,^^52^

To further help interpret our results, we link them to known processes that unfolded during the 2008 crisis: the crisis had a negative effect, especially on manufacturing employment from 2008 to 2013.^51^ It also unevenly affected men and women, where for men the negative impact was more acute.^56^ Also (heavy) industry employment tends to be more male-dominated and with more temporary employment^56^; for example, in the Territoire de Belfort in the East of France, the industry sector is the main user of temporary jobs (accounting for more than half) and nearly 3 out of 4 temporary jobs are held by men.^57^ From Figure 2G, this is reflected especially by the high PC1 coefficients in the Bourgogne Franche-Comté region which is the leading industrial region in France, as well as partly Grand Est (e.g., Haut-Rhin in Alsace). Among the dominant sectors are metallurgy, manufacturing of transport material, the agri-food industry, and manufacturing of rubber and plastic products.^58^ This can be seen in the employment intensity by sector and region, with highest values in the departments of Doubs and Jura in the Franche-Comté region (see Supplementary Note 2; this data is not directly used in our empirical analyses, but rather is complementary for explaining results). At the start of the crisis, this group of departments was not leading the dynamics of unemployment, but in the wake of the crisis, they superseded the departments in the mid-south and southeast. Although they do not show the highest unemployment rates (Figure 2C), they appear in the “vulnerable direction” (PC1), highlighting their slow recovery after the crisis and their susceptibility to future perturbations (see Supplementary Note 2).

At the onset of the crisis, in contrast, Corsica and Bouches-du-Rhône in the South of France were leading the unemployment dynamics (Figure 2F). In Corsica, tourism is a significant driver of the island’s economy, and with high construction employment intensity, particularly in Corse-du-Sud (Supplementary Note 2). Although Bouches-du-Rhône in the Provence-Alpes-Côte d'Azur region has a relatively more diversified economy than Corsica, tourism is still a key component of its economy; both rely heavily on tourism-related revenue.^58^

## Discussion

Countries are recurrently exposed to periods of economic crises. Improving the resilience of economic systems is of great policy and societal interest. Time series data and applying relevant metrics, such as autocorrelation and variance, EWS may reveal when resilience breaks down, but do not cover the spatial aspects of resilience and hence provide only limited information about the related mechanisms behind resilience. Spatiotemporal data may reveal spatial differences in time that may improve our understanding of how an economic crisis impacts the economy and how the economy recovers. Key questions we looked to answer in this article were whether more information about the resilience of the system is gained by considering the spatial dimension, and whether ecological indicators can be used as a detection method of impending economic crises. Based on our work, it seems it does, and that they can. In particular, we performed a spatial disaggregation of PCA, and calculated spatial autocorrelation as an SEWS on regional unemployment data. This revealed additional information on resilience that would otherwise be overlooked by using spatially aggregated data and univariate analyses. Even when an upcoming shift is unavoidable, knowing when the economy is losing resilience and which regions play an important role in the loss of resilience or lead the recovery of the economy is worthwhile. Either way, we argue for the use of spatially explicit data - if available - with SEWS when doing an economic resilience assessment.

Our findings suggest that different sectors are linked (in terms of dominance) to different regions, and that the sectors that dominate the unemployment signal pre-crisis (e.g., tourism) are different from the sectors dominating the signal after the crisis (e.g., heavy industry). The employees or investments are not necessarily exchangeable, which can explain why looking only at time series is insufficient: by only looking at the (aggregated) unemployment numbers, we overlook potentially important aspects of the ability to deal with or recover from economic disruptions. This is in line with Moro et al. (2021), who state that “the cities with adaptable labor markets are best prepared for the future of work” (p. 2).^12^ This also links to “response diversity” as a resilience mechanism,^54^ and relates to our findings of the shifting phenomenon via the spatially disaggregated PCA, which provides a signal that the system is losing resilience.^59^

To further evaluate the space-resilience link, we calculated Moran’s I as an SEWS based on critical slowing down. However, there is an alternative explanation for the link between Moran’s I and crises: when clustering is high, people living in an area cannot easily find a job somewhere outside of their home area, because it is likely that unemployment is high as well in those areas (e.g., if sectoral specialization is similar between contiguous spatial units). Therefore, our analysis does not prove that these moments of crisis are actual “tipping points,” but they do reveal a strong relationship between a strong increase in Moran’s I and an upcoming crisis. It also resembles the Panarchy framework, whereby increasing connectivity diminishes resilience, as in the case of a system-wide shock.^29^ Especially if there is a lack of an adjustment mechanism (e.g., via a spatial phenomenon such as migration), there can be an amplification of regional unemployment polarization. Since Moran’s I overall increasing trend makes it difficult to make strong conclusions, so we further checked its utility in signaling impending economic crises. Our results suggest that, besides the spatially disaggregated PCA, an SEWS approach may be useful for better understanding the resilience of economic systems to crises of different origins, such as the ones analyzed here.

### Limitations of the study

While this study provides useful insights to improve our understanding of economic resilience, complementing regional economic and economic geography work on the topic, there are limitations worth considering. Regarding the applicability to other contexts, the approach can be applied to other disaggregated data from other (non-) European countries as long as the numerosity of spatial units allows for meaningful inference. In the analysis, we use the unemployment rate as it is a seminal indicator of the state of an economy and is often used, either by itself or along with other labor market indicators, in the economic resilience literature – especially from a spatial perspective. However, an important aspect to further explore is the timescale of a variable when relating ecology to economics. Data of faster time-scales, such as prices, may expose other signals than unemployment, which is typically a slower-response variable. Also, there is room for more understanding of determinants affecting regions’ (lack of) resilience. This connects more to questions on causal processes and mechanisms. For example, the role of policies is an aspect to further explore, such as active labor market policies and whether they are or should be targeted to lower-performing regions and/or regions that are dominating the unemployment signal.

## Resource availability

### Lead contact

Further information and requests for resources and materials should be directed to and will be fulfilled by the lead contact, Sol Maria Halleck Vega (solmaria.halleckvega@wur.nl).

### Materials availability

This study did not generate new unique reagents.

### Data and code availability

• All data generated or analyzed during this study are included in this article. The data and sources in the supplementary information file are not used in the actual analyses carried out, but rather as a complement to explain some results. All data can be downloaded from the websites indicated in the key resources table.
• The MATLAB, Python, and R codes for the analyses are available at: https://github.com/elsweinans/french_unemployment_resilience. The results on the metrics presented in the figures within the article and its supplementary information files can be reproduced via the code.
• Any additional information required to reanalyze the data reported in this article are available from the lead contact upon request.

## Acknowledgments

The authors gratefully acknowledge the scientific editor, Dr. Camilla Imarisio, and three anonymous reviewers for their insightful and constructive suggestions.

## Author contributions

S.M.H.V., R.P., G.V., and E.W. contributed to the design, conceptualization, and writing – review and editing. S.M.H.V. and E.W. conducted the analyses. S.M.H.V. wrote the original draft. E.W. made the figures.

## Declaration of interests

The authors declare no competing interests.

## STAR★Methods

### Key resources table

**Table:** 

REAGENT or RESOURCE	SOURCE	IDENTIFIER
**Software and algorithms**		

MATLAB	The MathWorks Inc. (2022). Statistics and Machine Learning Toolbox Documentation, Natick, Massachusetts: The MathWorks Inc.	https://www.mathworks.com/help/stats/index.html
Python version 3.13	G. van Rossum, Python tutorial, Technical Report CS-R9526, Centrum voor Wiskunde en Informatica (CWI), Amsterdam, May 1995.	https://www.python.org
GeoPandas	Kelsey Jordahl, Joris Van den Bossche, Martin Fleischmann, Jacob Wasserman, James McBride, Jeffrey Gerard, … François Leblanc. (2020, July 15). geopandas/geopandas: v0.8.1 (Version v0.8.1).	https://doi.org/10.5281/zenodo.3946761
R	R Core Team (2021). R: A language and environment for statistical computing. R Foundation for Statistical Computing, Vienna, Austria.	https://www.R-project.org/
Custom code	Authors (github)	https://github.com/elsweinans/french_unemployment_resilience

**Other**		

Unemployment data at NUTS-3 (department) level	National Institute of Statistics and Economic Studies (INSEE)	https://www.insee.fr/en/accueil
Employed persons by sector and NUTS-3 region Working age population by NUTS-3 region	Eurostat regional database	[nama_10r_3empers] Employment (thousand persons) by NUTS 3 region [demo_r_pjanaggr3] Population on 1 January by broad age group, sex and NUTS 3 region
Net occupancy rate of bed-places in hotels and similar accommodation	Eurostat regional tourism statistics	https://ec.europa.eu/eurostat/databrowser/view/tour_occ_anor2/default/table?lang=EN
Share of activity sectors characteristics of tourism in total salaried employment	Statista	https://www.statista.com/statistics/766710/share-employment-activities-tourist-region-la-france/

### Method details

#### Unemployment data

We use data on unemployment rates (in percentage) in France at the NUTS-3 level of geographical aggregation. NUTS-3 stands for Nomenclature of Territorial Units for Statistics level 3, which is one of the official regional classification levels from Eurostat. The sample covers all departments in metropolitan France, and the data are produced by the National Institute of Statistics and Economic Studies (INSEE). The data had a time resolution of 3 months, meaning there were 4 datapoints per year, and spanned the period from Q1 1982 to Q2 2019.

### Quantification and statistical analysis

#### Principal component analysis

To disentangle the dynamics of the regional unemployment time series, we perform a Principal Component Analysis (PCA) on the temporal unemployment data. Literature on resilience indicators suggests that, as a system loses resilience, the system slows down.^59^ In multivariate systems, this critical slowing down may have a ‘direction’, i.e., there is a set of variables (in our case the variables represent regions) that, when perturbed together, recover slowly,^49^^,^^60^ whereas perturbations in other directions may not show any sign of critical slowing down.^61^ PCA allows us to capture this slow direction.

We follow the approach of Lever et al., (2020)^49^ and perform PCA on the unemployment data in a moving window of 10 years. Thus, for every 10 year-period, we identify the regions that are dominating the signal during that time (i.e., the regions that show larger and/or synchronized fluctuations within that period) by calculating the first principal component (PC). The regions that have high scores on the first PC are hence likely to play a role in the onset of the financial crisis. Furthermore, as the system is losing resilience and becoming more dominated by the dynamics on the first PC, the explained variance of the first PC will increase.^49^ We therefore plot the explained variance as calculated on the moving window PCA.

Apart from analyzing the dynamics of the first PC, we also look at the spatial pattern of our first PC. The scores of the first PC, reflect different regions’ contribution to the first PC. By visualizing the scores of the first PC on the map, regions are identified that reflect relevant industries related to the changes in employment. Therefore, we visualize the first PC on the map of France right before and right after the 2008 financial crisis, as the most important economic crisis within the available time series. This PCA was performed in Matlab, using the build-in pca() function, while spatial visualizations (i.e., Figures 2B, 2C, 2F, and 2G) were created in python using GeoPandas.^62^

#### Spatial early warning signals

To determine if the spatial signal can be used as an ‘early warning’ for the economic crisis, we calculate spatial autocorrelation (SA) for the regional unemployment rates. SA has been suggested as a spatial early warning signal (SEWS) in ecological systems, mainly in the context of vegetation patterning in semi-arid regions.^25^ Several ecological studies employ SA as a resilience metric (see Scheffer et al. (2015)^63^ for a comprehensive overview). SEWS have been advanced, for example, in seminal works of Dakos et al. (2010) and Kéfi et al. (2014).^25^^,^^50^ Here, SA is calculated via the Moran’s I (MI) statistic based on a first-order contiguity spatial weights matrix,^64^ as applied in ecological studies. This means that regions are considered as neighbors when they share a common border. As a system approaches a tipping point, it will become intrinsically slower and this can cause an increase in the short-term memory (correlation at low lag) prior to a transition.^50^^,^^61^ A binary contiguity specification is also commonly applied in spatial econometric studies, at times with variations of contiguity order or distance; non-geographical information (e.g., institutional proximity), is used more rarely since it can introduce endogeneity. When considering especially geographical-based matrices, it has been shown that changes in the spatial weights matrix do not lead to substantial differences in inferences.^65^^,^^66^ NUTS-3 regions are often used to analyze labor market characteristics, and can encompass commuting zones, as well as migration flows. In addition to following the SEWS literature, we take order one contiguity, as migration tends to be less pronounced in European regions compared to, for example, the United States. Even in the largest metropolitan area of Paris, specifying the matrix in this way covers a large part of this area (e.g. the surrounding departments of Val-de-Marne and Hautes-de Seine). Moran’s I values usually range between about –1 and +1, where values closer to –1 indicate negative SA, -1/(n-1) for no SA (i.e., randomly distributed spatially), and closer to 1 indicating positive SA. It has been shown theoretically and empirically that, especially if there is significant connectivity and spatial heterogeneity, SA can be a better indicator of an impending shift than solely time-series-based indicators. This is true for the biophysical systems that are studied, for instance, systems in which local plant-water interactions lead to spatial patterning that is picked up by an indicator based on SA. Sankaran et al. (2018)^67^ compared different SEWS, and found that SA at lag 1 produced fewer failed or misleading signals on an in-silico data set than some other SEWS. This analysis was performed in R.

To test when exactly the MI gave a warning signal, we used three criteria. First, we calculated when it would surpass more than one standard deviation above its three year average value, a method proposed in Quax et al. (2013).^19^ Second, we required the Kendall tau correlation of the past 3 years’ of MI values to be higher than 0.9. Third, we required the accompanying p-value of the Kendall tau to be below 0.05. When all three criteria were met, we considered our MI to give an early warning signal. These calculations and visualizations were performed in Matlab.
