# Supplementary material for: Spatial early warning signals to assess economic resilience
Source: iScience. 2025 Nov 17;28(12):114097. doi: 10.1016/j.isci.2025.114097 (PMC12722024; doi:10.1016/j.isci.2025.114097)
Supplement: Document S1. Figures S1–S3 and Tables S1–S2 [file mmc1.pdf]

iScience, Volume *28*

Supplemental information

Spatial early warning signals

to assess economic resilience

Sol Maria Halleck Vega, Roberto Patuelli, George van Voorn, and Els Weinans

Supplementary Information

Supplementary Note 1

Sensitivity analysis of Moran's I as an indicator in signaling impending economic crises.

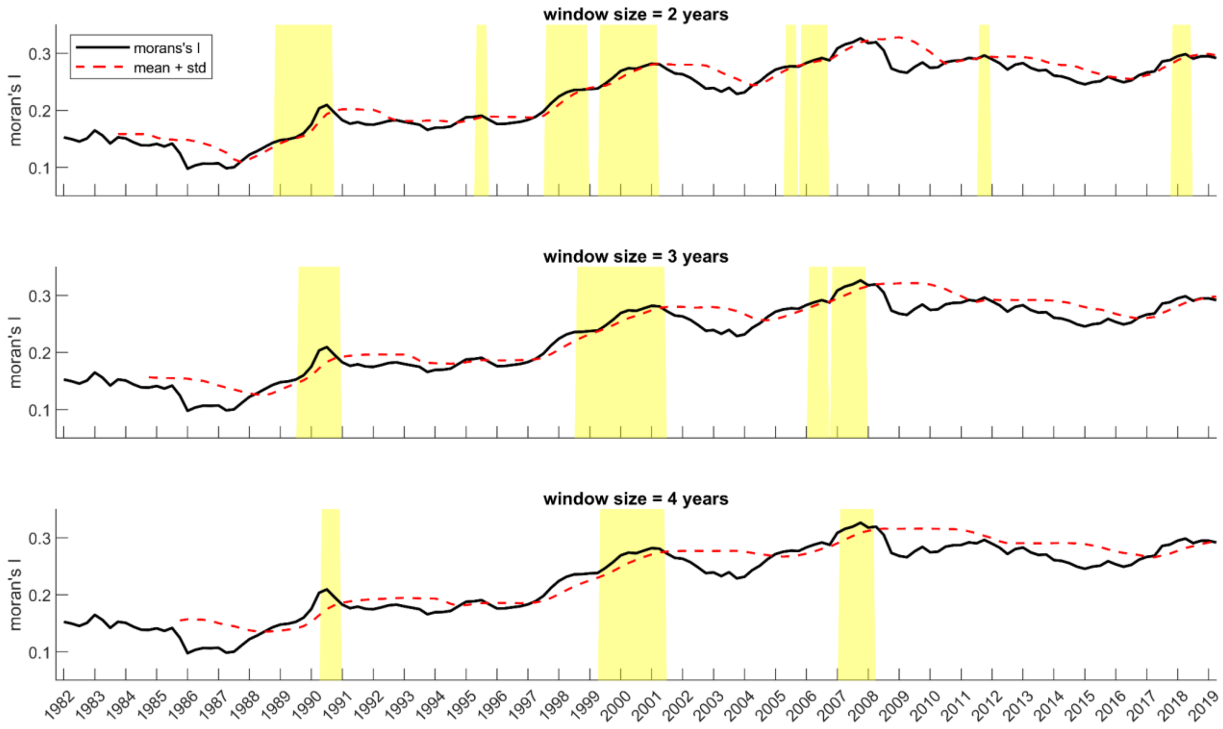

Fig. S1 Varying window size.

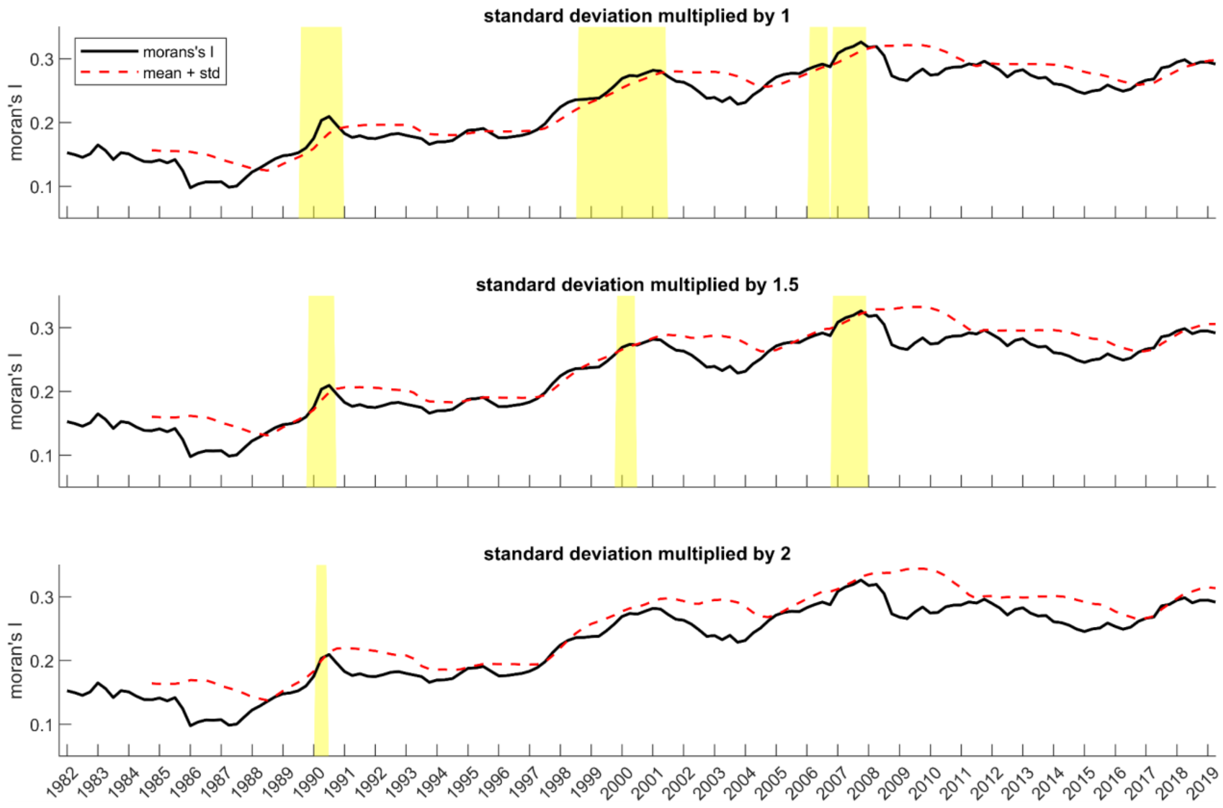

Fig. S2 Changing value multiplied by standard deviation.

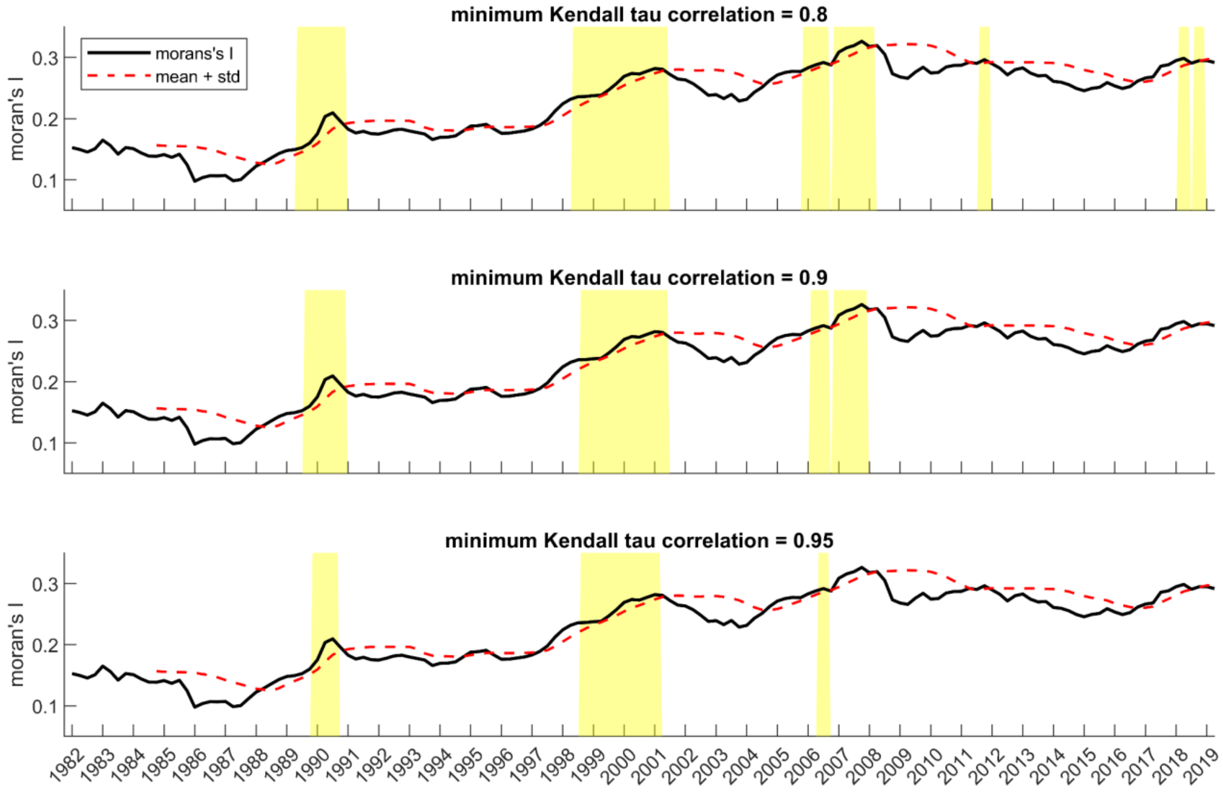

Fig. S3 Considering different Kendall tau correlations.

Supplementary Note 2

Key sector-related statistics

A)

	Employment intensity: Industry (except construction)								
	2007	2008	2009	2010	2011	2012	2013	2014	2015
**Franche-Comté**									
Doubs	13.2	12.9	12.1	11.7	11.9	11.6	11.4	11.3	11.2
Jura	13.1	12.8	12.3	12.0	11.8	11.8	11.8	11.7	11.6
Haute-Saône	11.1	10.9	10.2	9.8	9.8	9.8	9.7	9.7	9.6
Territoire de Belfort	10.7	11.1	10.3	9.8	9.5	9.0	8.9	8.8	8.7
**Alsace**	11.2	10.9	10.3	9.8	9.8	9.8	9.6	9.6	9.4
Haut-Rhin	11.8	11.4	10.8	10.1	10.0	10.0	9.8	9.6	9.4
Bas-Rhin	11.0	10.7	10.2	9.8	9.8	9.8	9.7	9.7	9.6
**Provence-Alpes-Côte d'Azur**									
Vaucluse	6.2	6.1	5.8	5.6	5.7	5.7	5.7	5.7	5.6
Bouches-du-Rhône	5.9	5.9	5.8	5.6	5.7	5.7	5.7	5.7	5.6
Alpes-de-Haute-Provence	5.0	5.2	5.2	5.0	5.0	5.2	5.2	5.3	5.4
Alpes-Maritimes	4.8	4.6	4.5	4.4	4.4	4.4	4.4	4.4	4.4
Var	3.5	3.5	3.3	3.3	3.3	3.3	3.4	3.3	3.3
Hautes-Alpes	3.0	3.2	3.0	2.9	2.8	2.8	2.8	2.8	2.9
**Languedoc-Roussillon**									
Gard	6.0	5.9	5.6	5.5	5.2	5.2	5.1	5.0	5.0
Lozère	5.0	4.7	5.2	5.4	5.4	5.7	5.7	6.0	6.0
Aude	3.9	3.8	3.7	3.5	3.5	3.5	3.5	3.6	3.5
Hérault	3.8	3.8	3.7	3.6	3.5	3.5	3.5	3.5	3.5
Pyrénées-Orientales	3.4	3.3	3.3	3.2	3.1	3.1	3.1	3.2	3.1
**Corsica**									
Haut-Corse	2.8	2.8	2.9	2.9	2.8	2.8	2.8	2.8	2.9
Corse-du-Sud	3.1	3.3	3.3	3.3	3.4	3.6	3.6	3.6	3.6
**National average**	8.7	8.5	8.1	7.8	7.7	7.7	7.6	7.6	7.5

B)

	Employment intensity: Manufacturing								
	2007	2008	2009	2010	2011	2012	2013	2014	2015
**Franche-Comté**									
Doubs	12.9	12.6	11.8	11.4	11.6	11.4	11.1	11.0	10.9
Jura	12.7	12.5	12.0	11.5	11.3	11.4	11.3	11.4	11.2
Haute-Saône	11.0	10.7	10.1	9.6	9.7	9.7	9.6	9.5	9.4
Territoire de Belfort	10.4	10.7	10.0	9.5	9.2	8.6	8.6	8.4	8.5
**Alsace**									
Haut-Rhin	11.1	10.7	10.1	9.4	9.4	9.4	9.1	9.0	8.7
Bas-Rhin	10.4	10.2	9.7	9.3	9.3	9.3	9.2	9.2	9.0
**Provence-Alpes-Côte d'Azur**									
Vaucluse	5.4	5.3	5.1	4.9	4.9	4.9	4.9	4.9	4.8
Bouches-du-Rhône	5.2	5.2	5.0	4.8	4.8	4.8	4.8	4.8	4.8
Alpes-de-Haute-Provence	4.2	4.3	4.4	4.2	4.2	4.4	4.4	4.5	4.6
Alpes-Maritimes	4.1	4.0	3.9	3.8	3.8	3.7	3.8	3.8	3.8
Var	2.9	3.0	2.8	2.7	2.7	2.7	2.8	2.8	2.7
Hautes-Alpes	2.4	2.4	2.3	2.2	2.1	2.0	2.1	2.1	2.2
**Languedoc-Roussillon**									
Gard	5.2	5.1	4.8	4.7	4.5	4.4	4.4	4.3	4.3
Lozère	4.3	3.9	4.5	4.8	4.8	4.8	5.1	5.1	5.2
Aude	3.3	3.3	3.1	3.0	3.0	3.0	2.9	2.9	2.9
Hérault	3.1	3.1	3.0	2.9	2.8	2.8	2.8	2.8	2.7
Pyrénées-Orientales	3.0	2.8	2.8	2.8	2.7	2.6	2.7	2.7	2.7
**Corsica**									
Haut-Corse	2.4	2.4	2.4	2.3	2.4	2.3	2.4	2.4	2.4
Corse-du-Sud	2.2	2.3	2.3	2.3	2.4	2.5	2.4	2.4	2.4
**National average**	8.0	7.8	7.4	7.1	7.0	7.0	6.9	6.9	6.8

C)

	Employment intensity: Construction								
	2007	2008	2009	2010	2011	2012	2013	2014	2015
**Franche-Comté**									
Doubs	4.1	4.1	4.1	4.0	4.0	4.0	4.0	3.9	3.8
Jura	4.6	4.6	4.6	4.6	4.5	4.4	4.4	4.5	4.4
Haute-Saône	4.0	4.1	4.1	4.0	3.9	3.7	3.6	3.6	3.5
Territoire de Belfort	3.5	3.6	3.5	3.4	3.4	3.4	3.2	3.1	3.2
**Alsace**									
Haut-Rhin	4.4	4.4	4.4	4.3	4.3	4.3	4.3	4.3	4.2
Bas-Rhin	4.4	4.5	4.5	4.5	4.4	4.4	4.4	4.4	4.2
**Provence-Alpes-Côte d'Azur**									
Vaucluse	5.4	5.5	5.6	5.5	5.4	5.4	5.4	5.3	5.3
Bouches-du-Rhône	4.2	4.4	4.4	4.3	4.4	4.4	4.4	4.4	4.3
Alpes-de-Haute-Provence	5.5	5.4	5.5	5.2	4.9	4.9	4.8	4.8	4.7
Alpes-Maritimes	5.2	5.3	5.3	5.2	5.1	5.1	5.2	5.0	5.0
Var	5.0	5.1	5.1	5.2	5.2	5.0	5.1	5.0	4.9
Hautes-Alpes	6.9	6.9	6.6	6.3	6.1	6.0	6.1	6.1	5.8
**Languedoc-Roussillon**									
Gard	4.8	4.8	4.7	4.7	4.6	4.6	4.4	4.4	4.4
Lozère	5.6	5.6	5.8	5.6	5.6	5.7	5.7	5.6	5.4
Aude	4.9	4.9	4.8	4.8	4.6	4.5	4.4	4.3	4.2
Hérault	4.9	5.0	4.9	4.7	4.7	4.5	4.4	4.4	4.3
Pyrénées-Orientales	5.3	5.2	5.0	4.9	4.9	4.9	4.9	4.7	4.6
**Corsica**									
Haut-Corse	6.3	6.7	7.0	6.9	6.9	7.0	7.0	6.8	6.5
Corse-du-Sud	7.5	8.1	8.2	8.1	8.3	8.5	8.3	8.3	7.9
**National average**	4.6	4.7	4.7	4.6	4.6	4.6	4.5	4.5	4.4

Table S1. Sectoral employment intensity at , NUTS-3 regions (departments) in France. A industry, **B** manufacturing, and **C** construction, according to NACE (Nomenclature of Economic Activities) European statistical classification. The leading regions of the dynamics at the onset of the 2008 crisis are highlighted in blue; the ones leading after are highlighted in yellow. The NUTS-2 regions (in bold font) are indicated above their respective departments (in regular font). The highest percentages are in green font. In panel A, Alsace had the second highest value until 2010; then, it was Pays-de-la-Loire, with the highest values in Vendée and Mayenne. Similarly, in panel B except from 2009 onwards. Source: Authors' calculation taking the ratio of employed persons and working age population (15-64) from the Eurostat regional database.

A)

	2012	2013	2014	2015
**Franche-Comté**	37.0	36.6	35.7	39.0
**Alsace**	45.2	46.0	46.6	46.0
**Provence-Alpes-Côte d'Azur**	48.5	47.2	46.5	48.0
**Languedoc-Roussillon**	43.5	42.7	41.4	42.0
**Corsica**	54.3	53.8	51.5	55.0
**National average**	43.1	42.4	41.7	42.8

B)

**Corsica**	11.6
**Provence-Alpes-Côte d'Azur**	9.3
**Ile-de-France**	9

Table S2. A Net occupancy rate of bed-places in hotels and similar accommodation (%) (NACE Rev. 2 activity I55.1) by NUTS-2 regions (data unavailable at NUTS-3 level)**.** See note under Table S1. 2012 is the earliest year available. The only higher numbers than Corsica are observed in Paris. Source: Eurostat regional tourism statistics. **B** Share of activity sectors characteristic of tourism in total salaried employment in France, 2017 (data unavailable at NUTS-3 level). Source: Statista 2023.
